# *LDOC1* silenced by cigarette exposure and involved in oral neoplastic transformation

**DOI:** 10.18632/oncotarget.4512

**Published:** 2015-07-10

**Authors:** Chia-Huei Lee, Kao-Lu Pan, Ya-Chu Tang, Ming-Hsien Tsai, Ann-Joy Cheng, Mei-Ya Shen, Ying-Min Cheng, Tze-Ta Huang, Pinpin Lin

**Affiliations:** ^1^ National Institute of Cancer Research, National Health Research Institutes, Taipei, Taiwan; ^2^ National Institutes of Environmental Health Sciences, National Health Research Institutes, Taipei, Taiwan; ^3^ Department of Medical Biotechnology and Laboratory Science, Chang Gung University, Taoyuan City, Taiwan; ^4^ Department of Oral Medicine, National Cheng Kung University, Tainan City, Taiwan

**Keywords:** oral squamous cell carcinoma (OSCC), leucine-zipper downregulated in cancer 1 (LDOC1), cigarette smoke condensate (CSC), DNA methylation, malignant transformation

## Abstract

Previously, we identified global epigenetic aberrations in smoking-associated oral squamous cell carcinoma (OSCC). We hypothesized that cigarette exposure triggers OSCC through alteration of the methylome of oral cells. Here we report that cigarette smoke condensate (CSC) significantly changes the genomic 5-methyldeoxycytidine content and nuclear accumulation of DNA methyltransferase 1 (DNMT1) and DNMT3A in human untransformed oral cells. By using integrated analysis of cDNA and methylation arrays of the smoking-associated dysplastic oral cell line and OSCC tumors, respectively, we identified four epigenetic targets—*UCHL1*, *GPX3*, *LXN*, and *LDOC1*—which may be silenced by cigarette. Results of quantitative methylation-specific PCR showed that among these four genes, *LDOC1* promoter was the most sensitive to CSC. *LDOC1* promoter hypermethylation and gene silencing followed 3 weeks of CSC treatment. *LDOC1* knockdown led to a proliferative response and acquired clonogenicity of untransformed oral cells. Immunohistochemistry showed that *LDOC1* was downregulated in 53.3% (8/15) and 57.1% (20/35) of premalignant oral tissues and early stage OSCCs, respectively, whereas 76.5% (13/17) of normal oral tissues showed high *LDOC1* expression. Furthermore, the microarray data showed that *LDOC1* expression had decreased in the lung tissues of current smokers compared with that in those of never smokers and had significantly decreased in the lung tumors of smokers compared with that in normal lung tissues. Our data suggest that CSC-induced promoter methylation may contribute to *LDOC1* downregulation, thereby conferring oncogenic features to oral cells. These findings also imply a tumor suppressor role of *LDOC1* in smoking-related malignancies such as OSCC and lung cancer.

## INTRODUCTION

Oral cancer, of which the vast majority of cases are oral squamous cell carcinoma (OSCC), is the major subtype of head and neck cancers. Epidemiologic studies have indicated that cigarette smoking might play a major role in the etiology of oral cancer [1–3]. Studies have estimated the etiological fraction of oral cancer in men attributable to smoking at 70% [4]. Using the mortality data from countries and regions such as Japan, Europe, Australasia, and North America, previous epidemiologic studies have indicated that the major etiological factor responsible for death from oral cancers is smoking followed by heavy alcohol consumption [5, 6]. Overall, these studies provide convincing evidence of an association between cigarette smoking and OSCC.

Promoter hypermethylation plays a crucial role in the inactivation of tumor suppressor genes during carcinogenesis. Accumulating evidence suggests that cigarette smoke might influence the development of various human diseases by inducing epigenetic changes. A genome-wide study revealed the loci of peripheral-blood DNA displaying differing methylation levels in current smokers, former smokers, and people who had never smoked [7]. By using pyrosequencing technology focused on DNA repetitive sequences and some tumor suppressor genes, Liu *et al*. demonstrated that cigarette smoke condensate (CSC) induces progressive global genomic hypomethylation and locoregional DNA hypermethylation in cultured human respiratory epithelia after incubation for up to 9 months [8]. Lin et al identified that the tobacco-specific carcinogen nicotine-derived nitrosamine ketone (NNK) induces the accumulation of DNA methyltransferase 1 (DNMT1) and the promoter hypermethylation of tumor suppressor genes, such as *FHIT*, *p16^INK4a^*, and *RARB*, in lung cancer patients [9]. Our previous study revealed global epigenetic aberrations in smoking-associated OSCC patients, and identified *BEX1* and *LDOC1* as 2 X-linked tumor suppressor genes with promoter methylated in 75% and 89% of OSCC tumor samples, respectively [10]. In this study, we investigated whether cigarette exposure induces profound epigenetic changes in oral cells, causing the silencing of tumor suppressor genes through promoter DNA methylation, which is involved in the development of oral cancer.

## RESULTS

### CSC exposure changes DNA methylation content of oral cells

**Figure 1:**
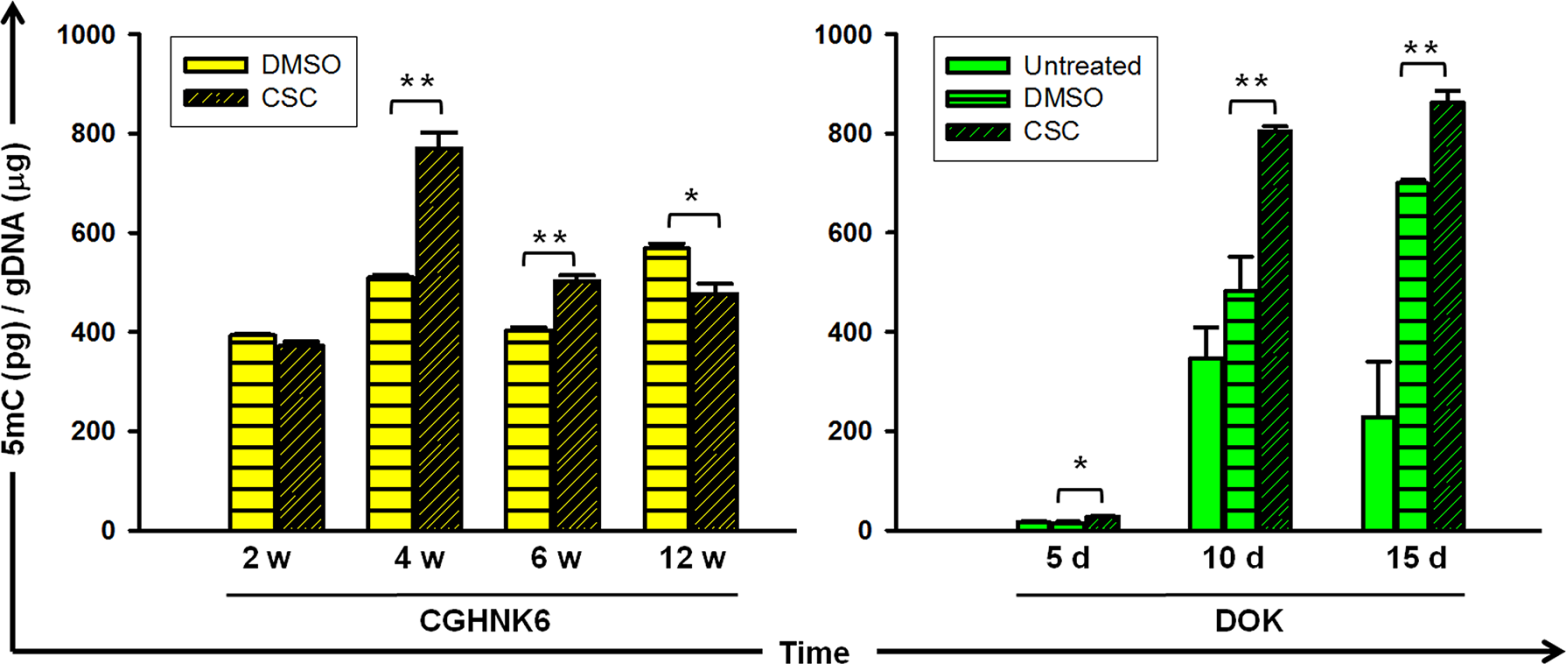
Genomic 5-methyl cytosine in oral cells with or without cigarette smoke exposure The amount of genomic 5mC was quantified using a commercial EIA kit in CGHNK6, and DOK cells. The treatment conditions for CGHNK6 and DOK were with or without of CSC (0.1 μg/mL) exposure for indicated time. DMSO-treated cells were used as vehicle controls for the CSC-treated cells. Data are presented as mean ± SD (*n* = 3). *p < 0.05 and **p < 0.01.

To determine the effects of smoking on the global DNA methylation content of oral cells, we measured genomic 5-methyl-2-deoxycytidine (5mC) in CGHNK6 (an immortalized untransformed oral keratinocyte cell line) [11] and DOK (a dysplastic oral keratinocyte derived from a heavy smoker with OSCC) [12] cells after CSC exposure by using an enzyme-linked immunoassay (EIA)-based method. The genomic 5mC content of CGHNK6 cells changed markedly, with a significant (*P* < 0.01) increase at 4 and 6 weeks, followed by a decrease (*P* < 0.05) at 12 weeks in the CSC-treated cells compared with that in the DMSO-treated (vehicle control) cells (Figure 1). The CSC treatment resulted in a significant (*P* < 0.01) increase in the genomic 5mC content at 10 and 15 days in the DOK cells compared with that in the untreated and vehicle control cells. These results suggested that cigarette smoking modifies the DNA methylation content of oral untransformed CGHNK6 or partially transformed DOK cells.

### CSC changes the nuclear accumulation of DNMT1 and DNMT3A in oral cells

**Figure 2:**
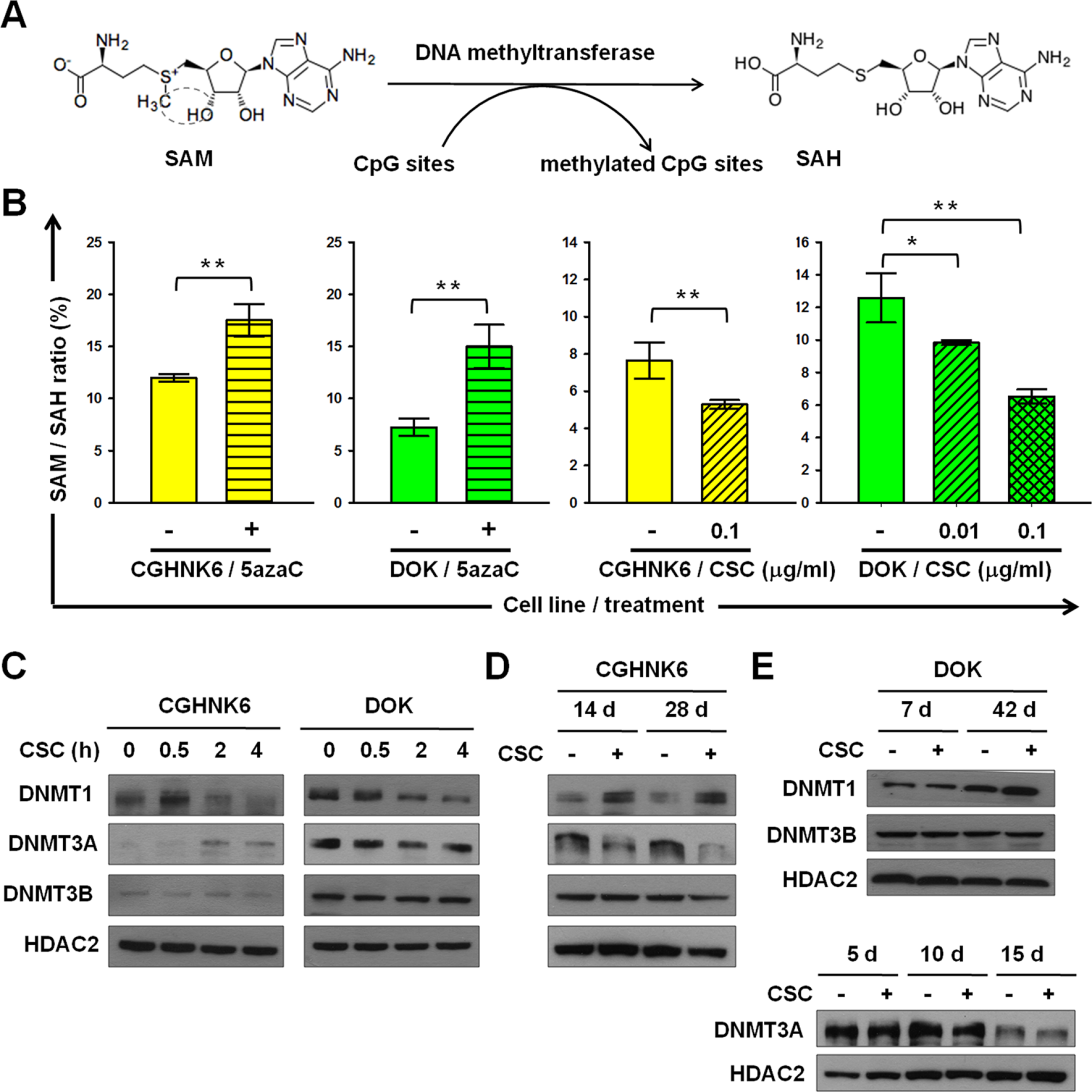
CSC changed the nuclear accumulation of DNMT1 and DNMT3A in CGHNK6 and DOK cells **A.** DNA methylation reaction catalyzed by DNMTs. **B.** The SAM/SAH ratios in CGHNK6 and DOK cells with or without 5aza-C treatment for 5 days and with or without CSC treatment for 3 weeks (CGHNK6) or 5 days (DOK). Data are presented as mean ± SD (*n* = 3). *p < 0.05 and **p < 0.01. **C, D.** and **E.** the amounts of nuclear DNMT1, DNMT3A, and DNMT3B were determined using western blotting at the indicated CSC (0.1 μg/ml) treatment times. DMSO-treated cells were used as vehicle controls.

S-adenosyl-methionine (SAM) is the major physiological methyl donor of DNMTs, including DNMT1, DNMT3A, and DNMT3B, which serve as the key enzymes in DNA methylation (Figure 2A). The ratio of intracellular SAM to its demethylated metabolite S-adenosyl-homocysteine (SAH) might provide an indirect indicator of DNMT activities, with an inverse correlation existing between the ratio of SAM/SAH and total DNMT activities. We established a liquid chromatography-electrospray ionization-tandem mass spectrometry (LC-ESI-MS/MS) platform with which to measure intracellular SAM/SAH ratios to assess the effects of cigarette smoke on DNMT activities. To evaluate the usefulness of our platform, we measured the SAM/SAH ratios of CGHNK6 and DOK cells with or without treatment by the DNA methyltransferase inhibitor 5-aza-dC (5azaC). The SAM/SAH ratio was significantly (*P* < 0.001) higher in the 5azaC-treated CGHNK6 and DOK cells than in the vehicle control cells (Figure 2B), indicating that the intracellular SAM/SAH ratio measured using the LC-ESI-MS/MS system provides a sensitive indirect indicator of cellular DNA methyltransferase activities. The SAM/SAH ratio of the CGHNK6 cells decreased significantly (*P* < 0.01) after CSC (0.1 μg/ml) exposure for 3 weeks (Figure 2B). The SAM/SAH ratio of DOK cells decreased in a dose-dependent manner by the CSC treatment for 5 days (Figure 2B). These results suggested that the activities of DNMTs may change in response to CSC exposure. Subsequently, we evaluated the effects of CSC on the nuclear accumulation of DNMT1, DNMT3A and DNMT3B. We conducted Western blot analyses with the nuclear fractions of cell lysates isolated from CSC-treated CGHNK6 and DOK cells. Regarding short-term exposure (Figure 2C), we observed that CSC treatment rapidly increased the nuclear accumulation of DNMT1 in the CGHNK6 and DOK cells within 0.5 hours and reduced the accumulation after 2 hours. The nuclear accumulation of DNMT3A slightly increased at 2 and 4 hours in the CSC-treated CGHNK6 cells, but decreased in the CSC-treated DOK cells during the experimental period (Figure 2C). Regarding long-term exposure, we observed that nuclear DNMT1 substantially increased in the CGHNK6 cells after 14 and 28 days of CSC treatment (Figure 2D). The amount of nuclear DNMT1 increased in both DMSO- and CSC-treated DOK cells after 42 days of incubation, with slightly higher levels in the CSC-treated cells (Figure 2E). The nuclear accumulation of DNMT3A decreased markedly after 14 and 28 days in the CSC-treated CGHNK6 cells compared with that in the vehicle controls (Figure 2D). The amount of nuclear DNMT3A decreased substantially in both the DMSO- and CSC-treated DOK cells after incubation for 15 days (Figure 2E). None of the cell lines exhibited any significant changes in the amount of nuclear DNMT3B after CSC treatment under any of the analysis conditions. These results suggested that the changes in the nuclear accumulation of DNMT1 and DNMT3A after CSC exposure may contribute to the altered methylome of oral cells.

### Identification of tumor suppressor genes epigenetically silenced by cigarette exposure in OSCC

**Figure 3:**
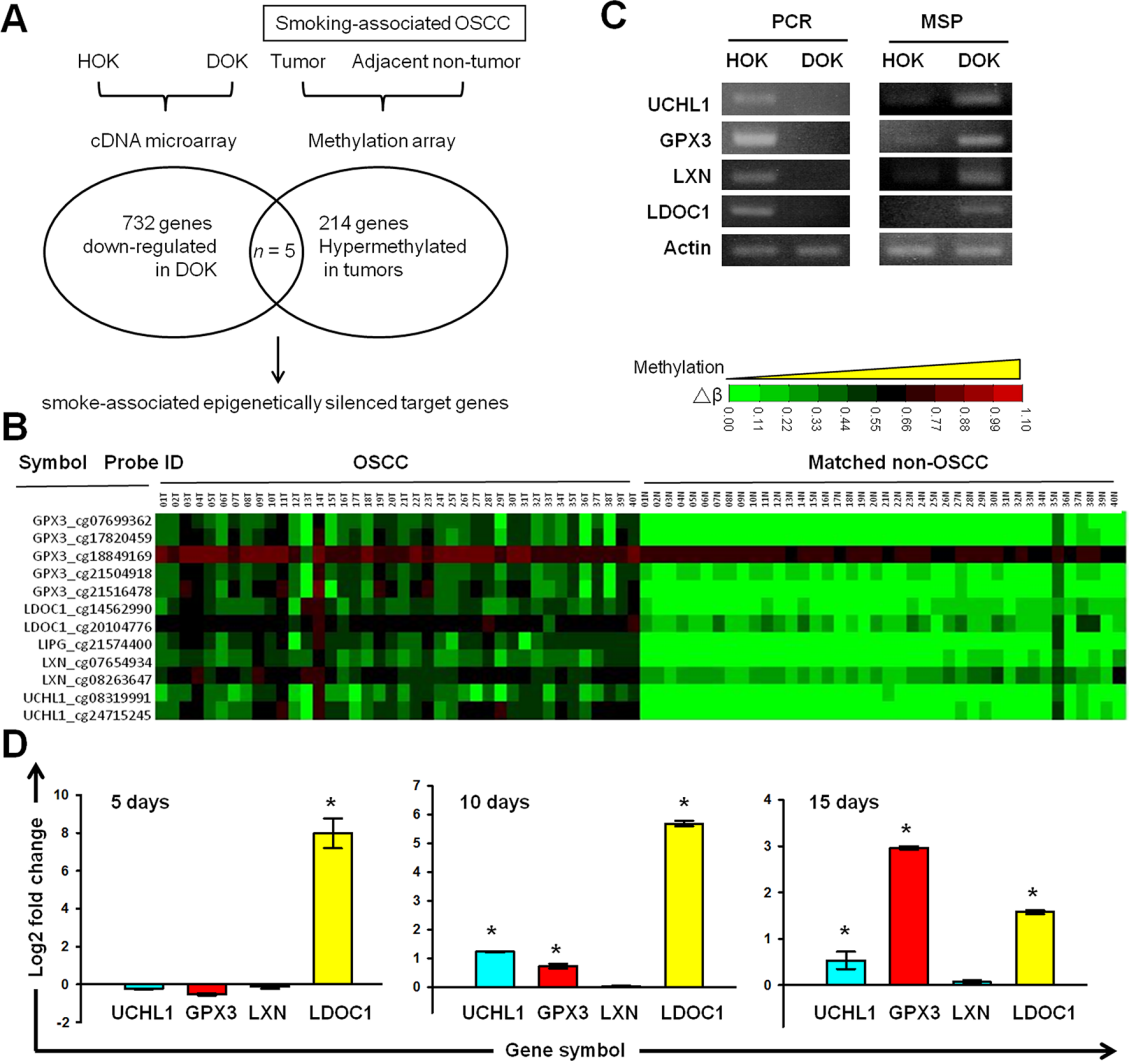
Identification of tumor suppressor genes silenced by cigarette exposure and DNA methylation in OSCC **A.** Strategy used for integrated microarray analysis. The methylation database (GEO Accession No. GSE38532) was established from the smoking-associated OSCC patients evaluated in our previous study [10]. **B.** Clinical methylation profiles of *GPX3*, *LDOC1*, *LIPG*, *LXN*, and *UCHL1* from tumor-nontumor pairwise smoking -associated OSCC. **C.** Gel images of qPCR and qMSP products from *UCHL1*, *LXN*, *GPX33*, and *LDOC1* in DOK and HOK cells. **D.** Effects of CSC exposure on the promoter methylation of *UCHL1*, *LXN*, *GPX33*, and *LDOC1*. DOK cells were treated with 0.1 μg/mL of CSC for 5, 10, and 15 days and then analyzed using a qMSP.

According to the smoking history of DOK's donor, the differentially expressed genes (DEGs) in DOK compared to normal human oral keratinocytes (HOK) may suggest a list of genes affected by long-term cigarette exposure and involved in the development of OSCC. The genes showing hypermethylation in tumors of smoking OSCC patients may include epigenetic targets important for smoking-related oral malignant transformation. To identify the potential epigenetically silencing tumor suppressor genes affected by cigarette smoke in OSCC, we conducted integrated analyses of the gene expression profiles obtained from the HOK and DOK, and the previously established methylation profiles of tumor-nontumor pairwise samples of smoking OSCC patients (Figure 3A). Using a Venn diagram analysis, we identified 5 genes (*GPX3*, *LDOC1*, *LIPG*, *LXN*, and *UCHL1*) with lower expression in DOK than in HOK, and increased methylation levels in OSCC tumors of smokers. Through a literature survey, we identified that *GPX3*, *LDOC1*, *LXN*, and *UCHL1*, but not *LIPG*, have been reported as tumor suppressor genes, and that their expression was mediated by promoter DNA methylation (Supplementary Table S1). We considered *GPX3*, *LDOC1*, *LXN*, and *UCHL1* as candidate tumor suppressor genes involved in oral malignant transformation and potentially silenced by cigarette exposure. Figure 3B displays the clinical methylation profiles of the *GPX3*, *LDOC1*, *LXN*, and *UCHL1* obtained from the 40 tumor-nontumor pairwise samples of smoking OSCC patients. We designed qPCR primers and promoter-located qMSP primers to validate our array data. Our results confirmed that *GPX3*, *LDOC1*, *LXN*, and *UCHL1* were lost expression and promoter methylated in the DOK, but expressed and promoter hypomethylated in the HOK (Figure 3C). To identify which gene among *GPX3*, *LDOC1*, *LXN*, and *UCHL1* was more susceptible to promoter methylation after CSC treatment, we conducted qMSP in the DOK cells after exposure to CSC for 5, 10, and 15 days. After CSC treatment for 5 days, *LDOC1* exhibited significant upregulation of promoter methylation, whereas the remaining three genes, *GPX3*, *LXN*, and *UCHL1*, exhibited no significant changes in promoter methylation (Figure 3D). The promoter methylation of *UCHL1* and *GPX3* was markedly increased after exposure to CSC for 10 and 15 days, respectively. We did not observe any promoter methylation of *LXN* in this time course qMSP study. These results suggested that the promoter methylation of *LDOC1* was more sensitive to CSC exposure than that of *GPX3*, *LXN*, and *UCHL1*. In a previous study, we identified *LDOC1* as an X-linked tumor suppressor gene epigenetically silenced in smoking-associated OSCC cases [10].

### *LDOC1* showed increased promoter methylation and silenced expression by CSC exposure in normal human oral cell line

**Figure 4:**
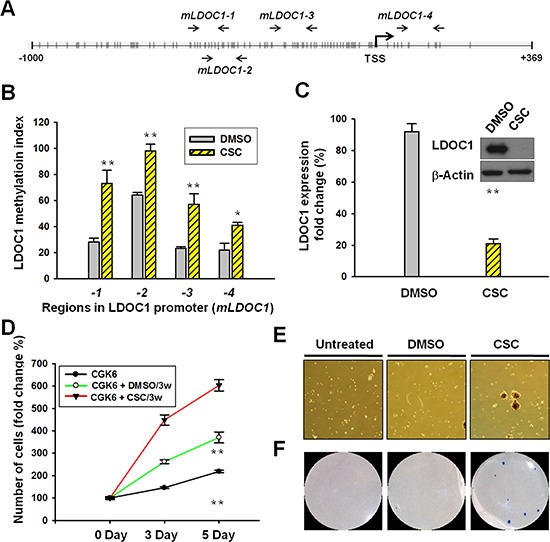
CSC treatment induced *LDOC1* silencing and promoter methylation in untransformed CGHNK6 cells accompanied by acquiring oncogenic properties **A.** Locations of qMSP primer pairs in the CpG island proximal to the *LDOC1* TSS. **B.** The methylation of CpG sites proximal to the *LDOC1* TSS was measured using qMSP. CGHNK6 cells treated with DMSO were used as vehicle controls. **C.** The expression of *LDOC1* mRNA (bar chart) and protein (gel images) was quantified using qPCR and western blotting, respectively, in CGHNK6 cells after exposure to CSC for 3 weeks or 6 weeks. The proliferation **D.** sphere-forming **E.** and anchorage-independent growth **F.** of CGHNK6 cells with or without CSC treatment (0.1 μg/ml) for 3 weeks. In proliferation assay, data are presented as mean ± SD (*n* = 3) analysed using the Student *t*-test. **p < 0.01.

Using Methyl Primer Expression software, we identified dense CpG sites in the *LDOC1* promoter region and exon 1 at positions −1186 to +369. We designed 4 pairs of qMSP primers mapping at regions *mLDOC1-1* (positions −567 to −460), *mLDOC1-2* (positions −542 to −417), *mLDOC1-3* (positions −354 to −227), and *mLDOC1-4* (positions −6 to 124) within the CpG island proximal to the transcriptional start site (TSS) of *LDOC1* (Figure 4A). The numbers of CpG sites in the 4 regions were 14, 15, 13, and 8, respectively (Table S2). Using these primers, we showed the CpG sites within the *LDOC1* promoter region were nearly fully methylated in DOK cells (Supplementary Figure S1). To examine the effect of CSC on *LDOC1* promoter methylation in untransformed oral cells, we conducted qMSP analysis with CSC-treated CGHNK6 cells. Results showed that the methylation of the regions covering the CpG sites proximal to the *LDOC1* TSS, *mLDOC1-1*, *mLDOC1-3*, and *mLDOC1-4*, increased progressively following exposure to CSC for 3 weeks and 6 weeks (Figure 4B), and the CpG sites mapped to *mLDOC1-2* were fully methylated within 3 weeks. As expected, increased promoter methylation was accompanied by the progressive downregulation of *LDOC1* mRNA expression following exposure to CSC for 3 weeks and 6 weeks (Figure 4C). Western blotting showed the LDOC1 proteins were barely detectable after 3 and 6 weeks of CSC treatment (Figure 4C). We did not analyze the effect of CSC exposure on *LDOC1* promoter methylation in other oral cell lines because *LDOC1* has already silenced by hypermethylation in all OSCC cells available [10]. Since the LDOC1 protein expression markedly reduced to a very low level by CSC treatment within 3 weeks, we examined whether the CSC exposure for 3 weeks confers CGHNK6 cells tumorigenicity *in vitro*. As shown in Figure 4D, the CGHNK6 cells markedly increased proliferation after CSC exposure for 3 weeks. DMSO- treatment for 3 weeks increased cell proliferation to a less extent. Oncogenicity was determined by the abilities of sphere-forming and anchorage-independent growth. CSC treatment for 3 weeks resulted in the acquisition of cancer cell phenotypes by showing sphere-forming (Figure 4E) and anchorage-independent growth (Figure 4F), whereas the DMSO treatment for 3 weeks did not confer CGHNK6 cells these transformed properties.

### *LDOC1* downregulation led to human untransformed oral cells acquiring clonogenicity and being associated with premalignant oral lesions and early stages of OSCC

**Figure 5:**
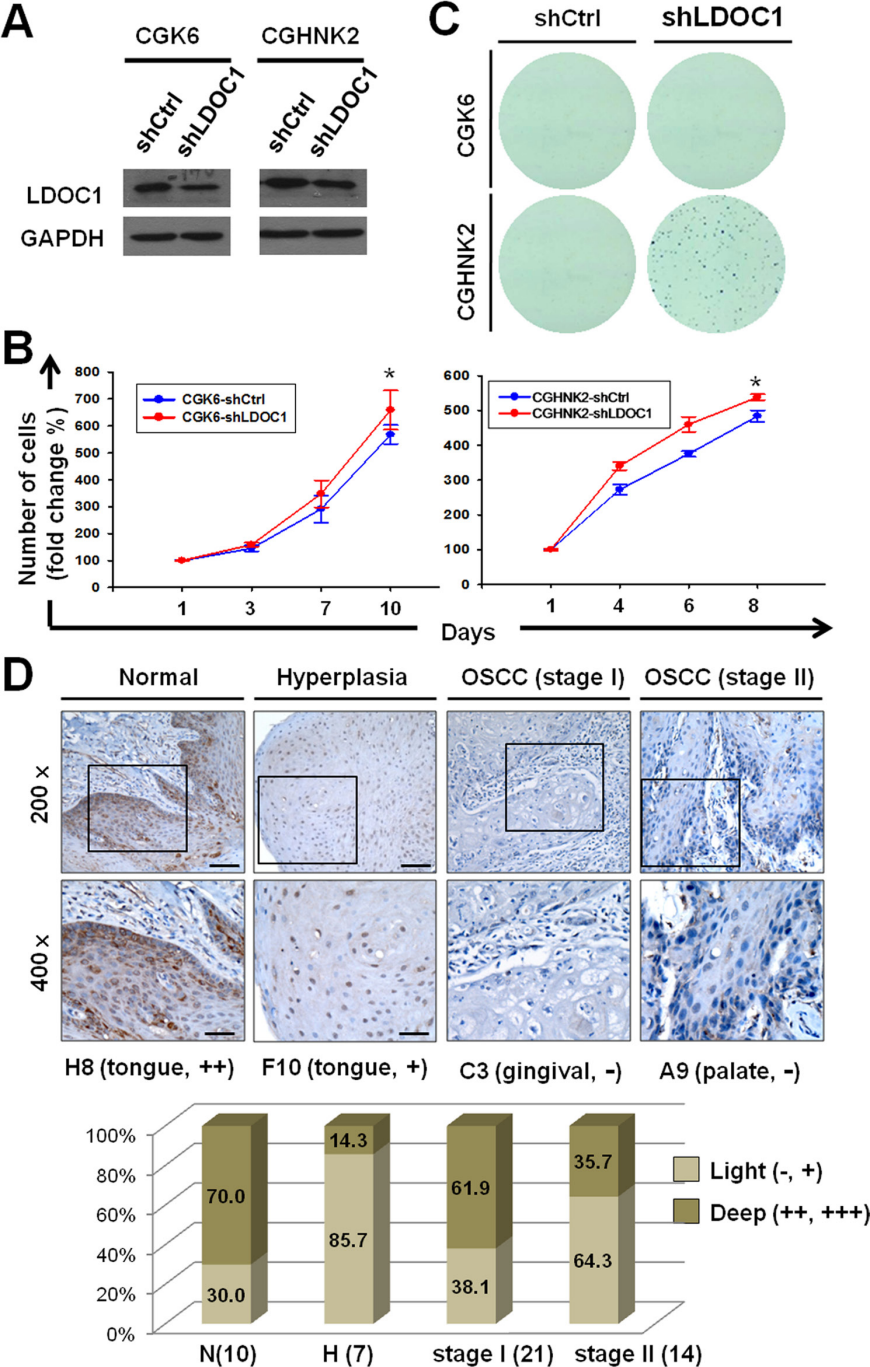
Downregulation of *LDOC1* was involved in oral neoplastic transformation **A.** Analysis of *LDOC1* mRNA and protein expression in CGHNK2 and CGHNK6 cells infected with lentivirus carrying sh*LDOC1* or shCtrl (vector control) by quantitative real-time PCR (lower) and Western blotting analysis (upper), respectively. **B.** *LDOC1* knockdown increased the cell proliferation of CGHNK6 (left) and CGHNK2 (right) cells. MTT assay was used to estimate the cell numbers at indicated time after seeding. Data are presented as mean ± SD (*n* = 3). *p < 0.05. **C.** Effect of *LDOC1* knockdown on anchorage-independent colony forming in CGHNK6 and CGHNK2 cells. **D.** The expression of LDOC1 protein in normal, diagnosed tissues of oral premalignant lesion (OPML), and early stages OSCC (stage I and II). Representative images for immunohistochemical analysis of LDOC protein expression were shown. The bar chart plots percentage of high or low LDOC1 protein expression in each group of oral samples.

To investigate whether *LDOC1* functions as a tumor suppressor gene in the early event of OSCC, we analyzed the effect of the downregulation of *LDOC1* on cell proliferation and oncogenicity of CGHNK6 and CGHNK2 (another untransformed oral keratinocyte cell line) [11] cells. We employed lentivirus-mediated shRNA method to reduce the expression of *LDOC1* in CGHNK6 and CGHNK2 cells. Cells were infected with lentivirus carrying shRNA specifically targeting *LDOC1*. The infection efficiencies were approximately 90%, as determined by detecting the expression of green fluorescent protein (GFP) 48 h after infection (Supplementary Figure S2). Puromycin was used for selection of puromycin-resistant cell pools. The mRNA and protein expression of *LDOC1* were analyzed by qPCR and Western blotting. The results showed that the *LDOC1* mRNA and protein significantly reduced in the CGHNK6-sh*LDOC1* and CGHNK2-sh*LDOC1* cells as compared to the corresponding control groups (CGHNK6-shCtrl and CGHNK2-shCtrl) (Figure 5A). To analyze the effect to the downregulation of *LDOC1* on cell proliferation, MTT assays were performed. As shown in Figure 5B, inhibition of *LDOC1* expression slightly but significantly increased the proliferation of CGHNK6 and CGHNK2 cells. To examine the effect of *LDOC1* on clonogenicity, soft agar colony formation assays were performed. The results showed that CGHNK6 cells with or without *LDOC1* knockdown (CGHNK6-sh*LDOC1* and CGHNK6-shCtrl) failed to form colonies (Figure 5C). Interestingly, CGHNK2-sh*LDOC1* cells formed numerous colonies whereas CGHNK2-shCtrl cells remained unable to grow colony on soft agar (Figure 5C). Giving that anchorage-independent growth as an important trait of malignant transformation, these results suggested that *LDOC1* downregulation not only induced cell proliferation but also drive the untransformed CGHNK2 cells toward carcinogenesis *in vitro*. These findings also implied that *LDOC1* downregulation may play an important role in OSCC. To examine the expression of LDOC1 protein in oral malignant transformation, we carried out immunohistochemistry study with tissue microarrays containing oral biopsies covering normal, precancerous lesions, and all stages of cancer progression. The result demonstrated LDOC1 expression was mainly located in the cytoplasm and 76.5% (13/17) normal oral tissues showed LDOC1 high expression, compared to 53.3% (8/15) oral premalignant lesions (OPML, including hyperplasia squamous epithelium and benign tumors) showing no or low LDOC1 expression (Figure 5D). Among 35 of early stage (21 for stage I and 14 for II) OSCC samples, the percentage of low LDOC1 expression were increased with increasing clinical stage, 47.6% (10/21) and 64.3% (9/14) for stage I and stage II OSCC, respectively (Figure 5D). However, 7 of 10 (70%) late stages (stage III and IV) OSCC showed high LDOC1 expression (data not shown), implying LDOC1 may play different roles in early and late stages of OSCC. Representative immunohistochemical results for LDOC1 in normal oral tissues, OPML, and OSCC were shown in Figure 5D. Altogether, these results suggested that *LDOC1* downregulation may promote tumorigenesis in early oral carcinogenesis.

### *LDOC1* downregulation was associated with the smoking status of non-cancerous and cancerous lung tissues

**Figure 6:**
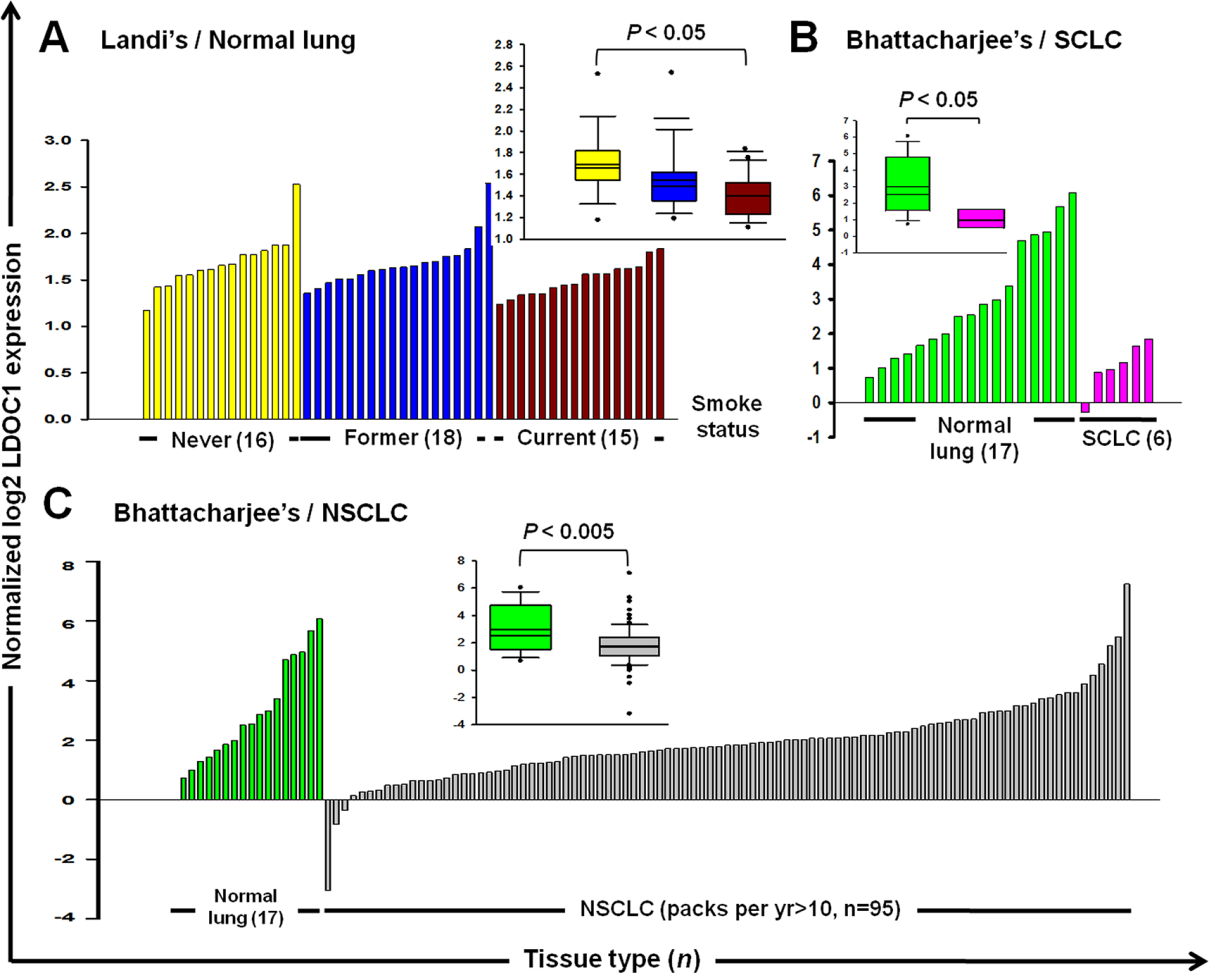
Downregulation of *LDOC1* in non-cancerous and cancerous lung tissues of smokers The *LDOC1* expression profiles of **A.** normal lung with information about smoking status [36], **B.** small cell lung carcinoma and **C.** and non-small cell lung carcinoma with smoking history [37], were obtained from publicly available microarray data sets in Oncomine (https://www.oncomine.com). Each of the normal and tumour samples is plotted in order of increasing levels of *LDOC1*. Inset: box plots display the median values of the array data and the 25^th^ and 75^th^ percentiles. The minimum and maximum values are indicated as whiskers. Points indicate outliers. Statistical differences were determined by Student *t* test.

Besides oral cavity, lung also frequently expose to cigarette smoke which has been well recognized as the major causative factor to lung cancer. Therefore, it is possible that *LDOC1* might decrease expression by cigarette exposure in lung of smokers and smoking-associated lung cancer. To test this, we performed a search of the publicly available microarray studies in the Oncomine database (www.oncomine.org) to examine the expression of *LDOC1* in normal and cancerous lung tissues of smokers and non-smokers. In Landi's study [13], *LDOC1* expression significantly decreased in lung tissues of 15 current smokers compared to those from 16 never smokers (Figure 6A). There were no differences in *LDOC1* expression between the former smokers (*n* = 18) and never smokers. In Bhattacharjee's study [14], *LDOC1* expression markedly downregulated in all of the 6 small-cell lung carcinoma (SCLC), the lung cancer type most strongly associated with cigarette exposure, compared to the normal lung tissues (without information of smoke status, *n* = 17) (Figure 6B). The same dataset also showed that *LDOC1* expression significantly decreased in tumors of non-small-cell lung carcinoma (NSCLC) patients with smoking habit (packs per year > 10, *n* = 95) as compared to normal lung (Figure 6C). These findings support the effect of cigarette smoke on *LDOC1* expression as well as a role of *LDOC1* in smoke-associated lung cancer.

## DISCUSSION

Cigarette contains numerous organic and inorganic carcinogens. Therefore, results from studies assessing the effects of single tobacco components, such as NNK or nicotine, might not reflect the comprehensive oncogenic effects of cigarette smoke. In this study, we prepared CSC according to a standard protocol and evaluated the epigenetic effects of CSC on oral cells. The effects of CSC on the methylome and DNMTs of oral untransformed CGHNK6 and partially transformed DOK cells were observed (Figures 1 and 2). Pickering's study [15] supports our findings indirectly. By whole-exome sequencing and copy-number analysis, Pickering et al [15] failed to observe any genetic mutation signature associated with smoking in oral tongue tumors. They proposed that the epigenetic alterations are possibly involved in the oncogenesis of smoke-associated squamous cell carcinoma of the tongue. SAM is the methyl donor for numerous methyltransferases. In biological systems, SAM is generated from methionine and donates its methyl group to various acceptor molecules, such as nucleic acids, lipids, and proteins, to be converted to SAH [16]. SAH can be recycled through the production of homocysteine and methionine. Thus, methyltransferase activity catalyzed by DNMTs plays a role in the homeostasis between SAM and SAH. In previous studies, the plasma concentration of SAH, not the SAM/SAH ratio, provided an indicator of DNA methylation status. Yi et al identified a positive correlation between plasma SAH levels and lymphocyte DNA hypomethylation in healthy young women [17]. Castro et al observed that patients with vascular disease displayed significantly higher plasma SAH concentrations, decreased plasma SAM/SAH ratios, and lower global DNA methylation statuses compared with control [18]. In animal models and in humans, the SAM/SAH ratio does not correlate with DNA methylation levels because it can be affected by various factors, such as methionine or folate in diets, physiological status, medical treatment, and numbers of methyl transferring enzymes [19, 20]. However, in our cultivated cell model, we limited the factors modulating the methylation reaction; therefore, the SAM/SAH ratio provided a sensitive indirect indicator of DNMT activities. We showed that 5azaC treatment results in a substantial increase in the SAM/SAH ratio of CGHNK6 and DOK cells (Figure 2B), supporting the efficacy of the SAM/SAH ratio in the assessment of DNMT activities. Previous studies have observed DNMT1 overexpression in patients with lung and liver cancers who were smokers [21, 22]. Lin *et al*. demonstrated that the tobacco-specific carcinogen NNK increases the stability of DNMT1, leading to its nuclear accumulation [9]. Consistent with this result, we observed a remarkable increase in nuclear DNMT1 in the CGHNK6 cells after exposure to CSC for 14 or 28 days (Figure 2D). In the CGHNK6 cells, DNMT3A was slightly increased after CSC treatment for 2 or 4 hours; however, it was markedly reduced after CSC treatment for 14 or 28 days (Figure 2D). Overall, our data suggested that changes in the nuclear accumulation of DNMT1 and DNMT3A may contribute to the aberrant methylome of untransformed oral cells exposed to cigarette smoke.

The DOK cell line was derived from tongue tissue with epithelial dysplasia adjacent to the OSCC tumor of a heavy smoker [12]. Considering the smoking history of the OSCC patient from whom the DOK cells were derived, we speculated that the DOK cell line could provide invaluable information on OSCC pathogenesis mediated by cigarette smoke. Using integrated array analysis, we identified 5 DNA methylation targets, including *GPX3*, *LDOC1*, *LIPG*, *LXN*, and *UCHL1* in the DOK and in a smoking OSCC patient cohort. Furthermore, we identified that the promoter methylation of *GPX3*, *LDOC1*, and *UCHL1* is sensitive to CSC exposure because significantly increased promoter methylation levels of these three genes were observed in the DOK cells within 15 days after CSC treatment (Figure 3D). *LDOC1*, an epigenetically silenced tumor suppressor gene in smoking-associated OSCC, was most sensitive to CSC and showed a remarkable increase in promoter methylation after CSC treatment for only 5 days (Figure 3D). Using CGHNK6 cells, we demonstrated the silencing of *LDOC1* expression and increased promoter methylation by CSC treatment within 3 weeks (Figure 4B and 4C). Additionally, we showed that the CpG islands within the promoter region of *LDOC1* were intensively methylated in smoker-derived DOK cells (Supplementary Figure S1). Altogether, these results suggest the methylation levels of *LDOC1* promoter are associated with cigarette smoke and may be used as a biomarker for tobacco exposure. Interestingly, the array data from lung tissues (Figure 6) provide supporting evidence for using *LDOC1* as an indicator for tobacco exposure. Landi's study [13] showed that, in normal lung, the expression of *LDOC1* was significantly decreased in lung of current smokers than that of never smokers (Figure 6A). Bhattacharjee's study [14] showed that the *LDOC1* expression was relatively low in small cell lung carcinoma—a lung cancer type strongly associated with smoking (Figure 6B). Although the relevant methylation levels of *LDOC1* for these studies were absent, the *LDOC1* down-regulation was likely caused by promoter methylation because we have demonstrated that the expression of *LDOC1* was majorly governed by promoter hypermethylation in our previous study [10]. In HOK and DOK cells, the methylation levels of *LDOC1* inversely correlated with the *LDOC1* mRNA expression (Figure 3C). We demonstrated that the methylation of *LDOC1* promoter increased accompanied by a reduction in *LDOC1* expression upon CSC treatment (Figure 4B and 4C). All these findings support that, in tissues of respiratory system, such as lung, which is frequently exposed to cigarette smoke, the expression and promoter methylation of *LDOC1* will be altered. Further investigation regarding the feasibility of using *LDOC1* as a molecular biomarker for cigarette smoke exposure by measuring the methylation of *LDOC1* in sputum samples is warranted.

We carried out shRNA-mediated *LDOC1* knockdown in the untransformed CGHNK6 and CGHNK2 cells to examine the tumor suppressor activity of *LDOC1*. Reduction in *LDOC1* expression led to acquired anchorage-independent growth of CGHNK2 cells whereas no colonies grew by the knockdown of *LDOC1* in CGHNK6 cells (Figure 5C). These differences in the tumorigenic potential driven by *LDOC1* downregulation may be due to some unidentified genetic differences between CGHNK6 and CGHNK2 cells. However, these results still suggest that downregulation of *LDOC1* may play a crucial role in the initiation stage of oral malignant transformation and contribute to the CSC-induced oncogenicity (Figure 4D–4F). In agreement with this hypothesis, the immunochemistry study revealed that *LDOC1* downregulation was frequently observed in tissues of OPML and the early stage of OSCC (Figure 5D). These results echo our previous study findings [10], which identified *LDOC1* as an X-linked tumor suppressor gene with promoter hypermethylation in 89% of OSCC patients who were habitual smokers. According to the tobacco-exposure sensitivity and *in vitro* anti-tumorigenic activity, we proposed that, in addition to oral cancer and lung cancer, *LDOC1* may function as a tumor suppressor gene and is downregulated in several human cancers associated with smoking. Publically available array dataset support our hypothesis. As shown in Supplementary Figure S3, the expression of *LDOC1* is also decreased in several smoking-associated cancers including cervical cancer [23], esophageal adenocarcinoma [24], pancreatic ductal adenocarcinoma [25], and head and neck cancers [26, 27]. Consistent with these array data, Nagasaki et al reported that *LDOC1* is a proapoptotic tumor suppressor gene in pancreatic cancer [28]. Using comparative proteomic analyses, Lui et al demonstrated the significant downregulation of *LDOC1* in serpin B5 (a tumor suppressor)-knockdown lung cancer cells [29]. Buchholtz et al further identified that *LDOC1* is frequently silenced by promoter hypermethylation in cervical cancer [30]. These findings support a potential etiological role of *LDOC1* in smoking-associated human cancers. The methylation status of *LDOC1* could potentially provide a molecular marker for the screening of smokers at high risk of cancer.

In summary, our findings not only strengthen the association between cigarette smoke and altered epigenome in OSCC, they also suggest a critical role of *LDOC1* in other tobacco-related cancers.

## MATERIALS AND METHODS

### Chemicals

*S*-(5′-Adenosyl)-*L*-methionine chloride dihydrochloride (SAM chloride dihydrochloride), and *S*-(5′-Adenosyl)-*L*-homocysteine crystalline (SAH crystalline), 5-aza-2-deoxycytidine (5-aza-dC) were purchased from Sigma-Aldrich. *S*-Adenosyl-*L*-methionine-*d_3_* (*S*-methyl-*d_3_*) tetra (*p*-toluenesulfonate) (*d_3_*-SAM) salt was purchased from C/D/N Isotopes Inc, acetic acid from J.T. Baker, acetonitrile and methanol were HPLC grade from Merck, water from Millipore.

### Cell lines and cell culture

Human oral keratinocytes (HOK) were obtained from the ScienCell and grown in Oral Keratinocyte Medium (OKM) with supplements (ScienCell). Immortalized untransformed human oral cell lines CGHNK6 and CGHNK2 was gift from Dr. Cheng (Chang Gung University, Taiwan) and grown in keratinocyte serum-free medium (KSFM) with supplements (Gibco) as previously described [11]. Briefly, CGHNK2, and CGHNK6 were primary culture cells from tissue biopsies of grossly normal oral mucosa with human papilloma virus (HPV) immortalization. Both cell lines were derived from patients with OSCC with the habits of smoking habit [11]. Human dysplastic oral keratinocyte DOK cell line were derived from dysplastic tissues neighboring to a tumor of an OSCC patient who was a heavy smoker before diagnosed as OSCC [12]. DOK cells were maintained in DMEM supplemented with 10% fetal calf serum, 100 g/ml penicillin, and 100 g/ml streptomycin.

### Preparation and treatment of cigarette smoke condensates (CSC)

CSCs were generated from Kentucky Reference Cigarettes 3R4F (University of Kentucky, Tobacco and Health Research Institute, Lexington, KY, USA) using a home-made smoking machine resembling that used by Pieraccini et al. [31]. Smoking was performed following a standard procedure agreed internationally by organizations such as the International Standards Organization and USA Federal Trade Commission [32]. “The modified Cambridge Filter method ultimately adopted by the Commission” is often referred to as the “FTC method”. The smoke condensates were trapped on glass fiber filters, weighed, and dissolved in DMSO according to the required concentrations. DOK and CGHNK6 cells were constantly treated CSC at indicated dose; and fresh medium containing CSC was replaced every 3 days. The DMSO-treated cells were used as controls. At appropriate times, cells were harvested, and processed for further analysis.

### Quantification of global DNA methylation

The global methylation of DNA was determined using competitive Enzyme Immunoassay (EIA) based commercial kit (Cayman's DNA Methylation EIA Kit), as the manufacturer's protocol. This assay is based on the competition between 5-methyl-2-deoxycytidine (5mC) in the sample and a 5mC-acetylcholinesterase (AChE) conjugate (5mC tracer) for a limited amount of 5mC monoclonal antibody (mAb). The amount of 5 mC tracer that is able to bind to the 5mC EIA mAb will be inversely proportional to the concentration of 5mC in the sample. The amount of mAb-5mC tracer complex binding to goat polyclonal anti-mouse IgG-coated ELISA plate was quantified by Ellman's reagent with a distinct yellow color and absorbs strongly at 412 nm. The relative global DNA methylation content of sample was obtained from calculating the amount of 5mC in the sample relative to those of CpGenome Universal Methylated DNA (Chemicon), a commercial fully CpG methylated DNA. All samples were analyzed in biological and technical duplicates.

### Intracellular SAM and SAH analysis

Intracellular metabolites extraction as described previously [33]. Liquid chromatography-electrospray ionization-tandem mass spectrometry (LC-ESI-MS/MS) consist of a PE series 200 binary pump and autosampler (PE series 200, Perkin-Elmer) system coupled with an API 3000 triple quadruple tandem mass spectrometer (Applied Biosystem). Liquid Chromatography separation against SAM, SAH, and *d_3_*-SAM were performed on an Atiantis T3 3um (2.1 mm i.d. × 150 mm) reversed phase column (Waters) at a flow rate of 250 uL/min. The compositions of mobile phase A and B are 0.1% acetic acid and 0.1% acetic acid in acetonitrile, respectively. The gradient profile were 0–3 min linear increase from 0 to 20% B, 3–6 min linear increase from 20 to 100% B, hold at 100% B for 3 min, 9–9.1 min from 100 to 0% B and hold at 0% B for 2.9 min. The detection of SAM, SAH and *d_3_*-SAM was carried out using positive electro-spray ionization (ESI) interface mode in a multiple reactions monitoring (MRM) scan mode. The MRM parameters of SAM, SAH and *d_3_*-SAM, were optimized: declustering potential (DP): 26, 31 and 61 V for SAM, SAH and *d_3_*-SAM, respectively, focusing Potential (FP): 190, 270 and 340 V for SAM, SAH and *d_3_*-SAM, respectively, collision energy (CE): 23, 29 and 23 V for SAM, SAH and *d_3_*-SAM, respectively, Collision Cell Exit Potential (CXP): 20, 8 and 16 V for SAM, SAH and *d_3_*-SAM, respectively.

### Extraction of DNA and RNA and bisulfite conversion of genomic DNA

Genomic DNA and total RNA from each sample were extracted using a QIAamp DNA mini Kit (Qiagen) and an RNeasy mini kit (Qiagen), respectively, according to the manufacturer's instructions. The extracted genomic DNA and total RNA were quantified and confirmed for OD 260/280 values between 1.8 and 2.2 and OD 260/230 values greater than 1. For methylation experiments, genomic DNA was bisulfite converted using BisulFlash DNA modification kit (Epigentek) according to the manufacturer's protocols. The converted DNA was stored at −80°C until ready to use.

### Expression microarray analysis

The Illumina HumanHT-12_V4 Expression BeadChip was used for genome-wide expression studies with HOK and DOK cells. Data analysis was performed with GeneSpring GX software (Agilent Technologies). Quantile normalization was performed. The microarray data used in this study have been deposited in NCBIs Gene Expression Omnibus (GEO) http://www.ncbi.nlm.nih.gov/geo/ and can be accessed through GEO Series accession number GSE54861 and GSE38532 [10].

### Quantitative real-time qPCR (qPCR)

The synthesis of cDNA from total RNA was performed with M-MLV reverse transcriptase (Promega). The primers required were designed by the online tool at the Universal ProbeLibrary Assay Design Center (Roche Applied Science, https://www.roche-applied-science.com) and shown in Supplementary Table S3. QPCR was performed with FastStart Universal Probe Master Kit (Roche Applied Science). The cycling parameters began with 95°C for 10 min, followed by 40 cycles of 95°C for 15 sec and 60°C for 60 sec, followed by a melting curve analysis.

### Real-time quantitative methylation-specific PCR (qMSP)

The bisultife converted DNAs were subjected to qMSP using the primers shown in the Supplementary Table S2 and S4. A primer pair specific for the CpG-free genomic region of the *ACTB* gene was used for normalization: 5′-TGGTGATGGAGGAGGTTTAGTAAGT-3′ (F) and 5′-AACCAATAAAACCTACTCCTCCCTTAA-3′. CpGenome Universal CpG Methylated and Unmethylated DNA (Chemicon) was used as positive and negative control, respectively. QMSP was performed using SYBR Advantage qPCR Premix (Clontech) as described previously [10].

### Knockdown of *LDOC1* in CGHNK6 and CGHNK2 cells

For knockdown of *LDOC1* expression, we purchased GIPZ lentivirus particles carrying shRNA specifically targeting *LDOC1* from Dharmacon. To cell infection, 50% confluent of CGHNK6 and CGHNK2 cells were incubated with lentivirus for 24 h, and the medium containing puromycin (5 μg/ml; Sigma-Aldrich) was replaced to select stable cell pools for at least 2 weeks before usage. Cells infected with lentivirus with empty vector (shCtrl) were used as controls.

### Proliferation assay

Cell proliferation was measured by the MMT [3-(4, 5-dimethylthiazol-2-yl)-2, 5-diphenyl tetrazolium bromide] assay. Cells were seeded at 5 × 10^3^ per well on 96-well plates. 10 μl of MMT (5 mg/ml) was added to each well at indicated time; the cells were further incubated for 4 h at 37°C and then subcultured in the medium with 100 μl of DMSO. The absorbance at 570 nm was measured on a micro ELISA reader (Bio-Rad).

### Soft agar assay

Soft agar assay was performed as described previously [34]. Briefly, cells were resuspended in culture medium containing 0.3% agarose at a density of 5 × 10^3^ cells in 6-well plates, then overlaid with 0.6% agarose and fed with fresh medium weekly for 3 weeks. After fixation and staining with violet blue, colonies were photographed and quantified using MetaMorph software (Molecular Devices). The experiment was performed three times, each time with four replicates.

### Immunohistochemistry

Tissue microarray covering tissue sections of oral cavity carcinoma progression, OR802 (10 cancer adjacent tissues or normal tissues, 7 hyperplasia epithelia, 8 benign tumors, and 45 oral carcinoma) were purchased from US BIOMAX. Immunohistochemistry was performed as previously described [35]. Briefly, all paraffin sections were dewaxed and rehydration followed by heating in a 0.01 M citrate buffer for 20 min. Subsequently, anti-LDOC1 (1:2000 dilution, LifeSpan, Newton, MA, USA) was incubated overnight at room temperature. The secondary biotinylated antibody and the streptavidin–peroxidase conjugate (BioGenex, Netherlands) were then incubated on the sections for each 20 min. The sections were stained with diaminobenzidine (DakoCytomation) for detection and stained with hematoxylin QS for counterstaining (Vector, Burlingame). Normal serum and phosphate buffer instead of specific antibodies was used as negative controls. Immunoreactivity was scored by the staining intensity using MetaMorph software (Molecular Devices). Staining intensities < 0.15 was described to be low LDOC1 protein expression, staining intensities > or = 0.15 was described to be high LDOC1 protein expression.

### Statistical analysis

Comparison of the results between various experimentally treated groups and their corresponding controls was carried out by Student's *t*-test. All comparisons were considered significant when *p* < 0.05.

## SUPPLEMENTARY MATERIAL FIGURES AND TABLES


