# Supplementary material for: LDOC1 silenced by cigarette exposure and involved in oral neoplastic transformation
Source: Oncotarget. 2015 Jul 10;6(28):25188–201. doi: 10.18632/oncotarget.4512 (PMC4694824; doi:10.18632/oncotarget.4512)
Supplement: Supplementary file 1 [file oncotarget-06-25188-s001.pdf]

SUPPLEMENTARY FIGURES AND TABLES

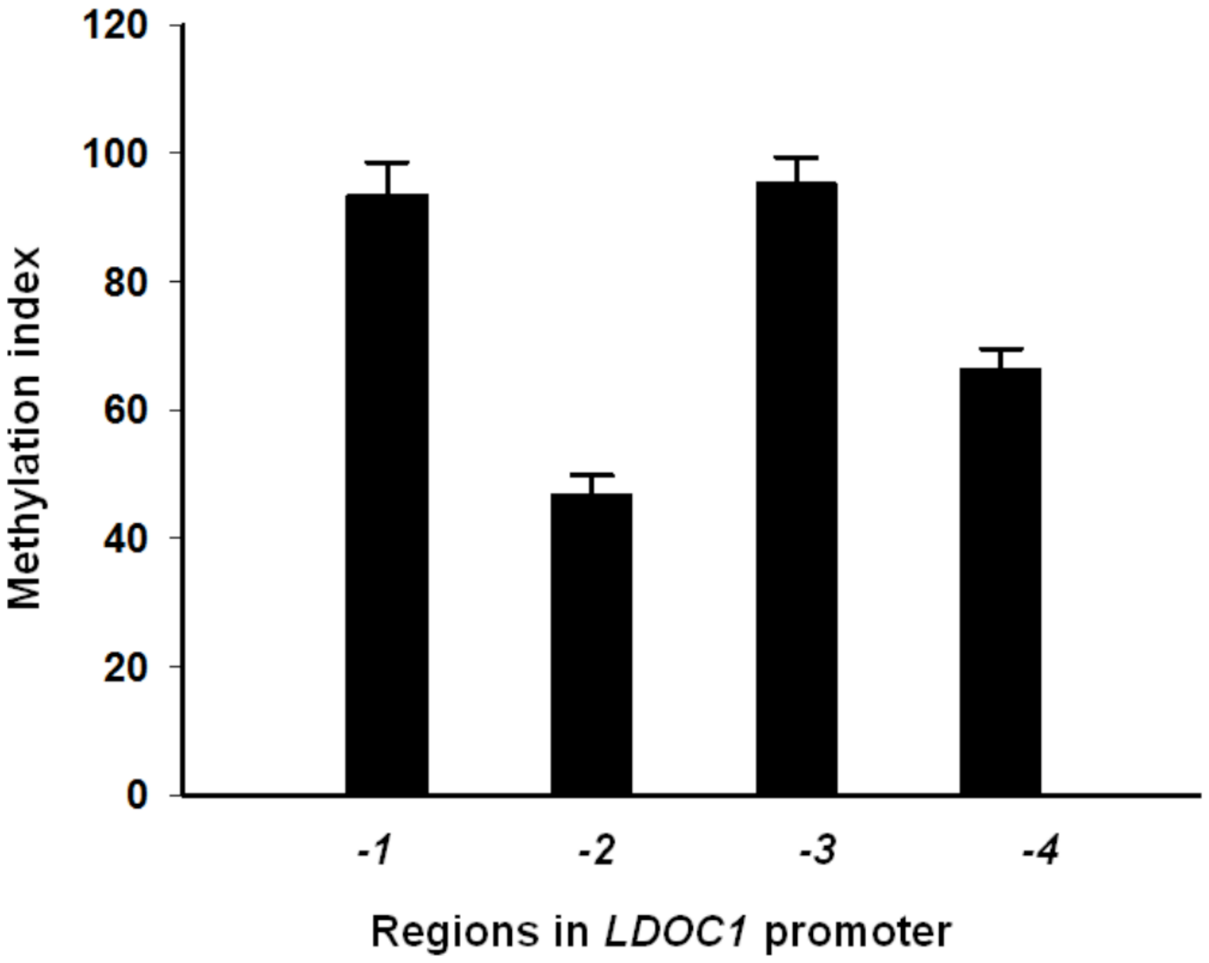

Supplementary Figure S1: The methylation profile of *LDOC1* promoter in DOK cells. The methylation level of *LDOC1* in the commercial fully CpG methylated genomic DNA was set as 100%.

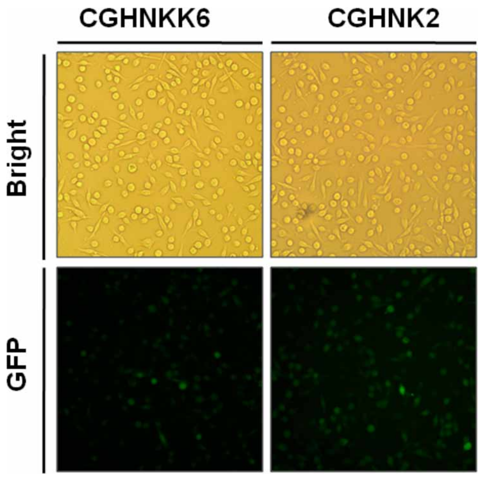

Supplementary Figure S2: Detection of lentivirus infection efficiency. The CGHNK6 and CGHNK2 cells were infected with lentivirus carrying *LDOC1* shRNA, and phase contrast (upper) or GFP (lower) images were obtained 48 h after infection. Magnification: 100 ×.

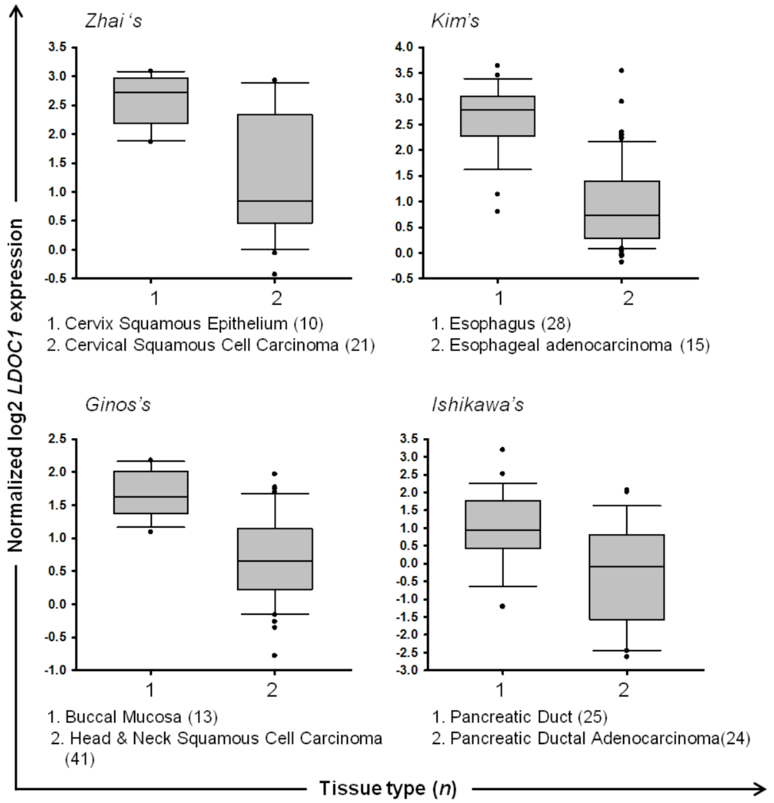

Supplementary Figure S3: Downregulation of *LDOC1* in smoking-associated human cancers.

Supplementary Table S1: Gene targets might silenced by cigarette exposure and promoter hypermethylation in OSCC

Accession	Symbol	Gene description	Log Fold	Literature survey	
			DOK vs HOK	Methylation	TSG[c]
NM_004181	*UCHL1*	Ubiquitin carboxyl-terminal esterase L1	–2.5759306	ˇ	ˇ
NM_020169	*LXN*	Latexin	–1.1163867	ˇ	ˇ
NM_006033	*LIPG*	Lipase, endothelial	–1.2515564		
NM_002084	*GPX3*	Glutathione peroxidase 3	–2.00635	ˇ	ˇ
NM_012317	*LDOC1*	Leucine zipper, down-regulated in cancer 1	–2.9763725	ˇ	ˇ

Supplementary Table S2: Information of qMSP primers for *LDOC1* promoter

Regions	Primers	Sequence 5′-3′	Location[a]	Amplicom (bp)	# CpG[b]
mLDOC1-1	mLDOC1-4F	5′ CGGTGTTTTTTTTCGAAAC 3′	–460	108	14
	mLDOC1-4R	5′ CGTCGTTACAAAATCGATTC 3′	–567		
mLDOC1-2	mLDOC1-3F	5′ CGAGAGGGTTAGTTTGTTGC 3′	–417	126	15
	mLDOC1-3R	5′ ACGAACATATAAAACGCCGT 3′	–542		
mLDOC1-3	mLDOC1-2F	5′ CGTGGATACGTATTTTTTGC 3′	–227	128	13
	mLDOC1-2R	5′ CATCAAAAACGCGTACAAC 3′	–354		
mLDOC1-4	mLDOC1-1F	5′ TGAGATCGGATTTAGCGTTC 3′	–6	130	8
	mLDOC1-1R	5′ CTAAATCGCTAAACCACCGA 3′	124		

[a]The 5′ nucleotide number where the primer starts (from transcription start site).
[b]Amount of the CpG dinucleotides.

Supplementary Table S3: Information of qPCR primers for *UCHL1*, *LDOC1*, *LXN*, and *GPX3*

Symbol	Primers	Sequence 5′-3′
UCHL1	UCHL1-PF	AGATCAACCCCGAGATGCT
	UCHL1-PR	ACCGAGCCCAGAGACTCC
LDOC1	LDOC1-PF	5 -ATGACGACGAAGACGACGA-3
	LDOC1-PR	5 -GAGGGTCGAGGGCCTAATAA-3
LXN	LXN-PF	AAACAGCAGCTTCCGCACT
	LXN-PR	AGTCGCAAGCTCCTTCAGTC
GPX3	GPX3-PF	GGGGACAAGAGAAGTCGAAGA
	GPX3-PR	GCCAGCATACTGCTTGAAGG

Supplementary Table S4: Information of qMSP primers located in promoter CpG islands of *UCHL1*, *LDOC1*, *LXN*, and *GPX3*

Symbol	Primers	Sequence 5′-3′
UCHL1	UCHL1-MF	5′ GTATTTTGGGAGGTCGAGAC 3′
	UCHL1-MR	5′ ACTAAAACTACAAACGCCCG 3′
LDOC1	LDOC1-MF	5′ GAGATCGGATTTAGCGTTC 3′
	LDOC1-MR	5′ AATACGCTAAATCGCTAAACC 3′
LXN	LXN-MF	5′ AGTTGGGATTATAGGCGTTC 3′
	LXN-MR	5′ CAACGCAAATAAATCACGA 3′
GPX3	GPX3-MF	5′ AGACGGAGTTTCGTTTTGTC 3′
	GPX3-MR	5′ AAATTAACCGAACGCGATAA 3′
